# The Nursing Supplement

**Published:** 1888-08-11

**Authors:** 


### The Nursing Mirror.

All Contributions for this Supplement should be addressed "The Nursing Mirror," care of The Editor, 38, Old Jewry, E.C.

### En Passant.

FRENCH PHRASES.—We think the following phrase is rather fine, even for a French woman and an editress: "I prefer the ambulance to the tribune, and all I demand of the Revolution is my share of devotion and danger." It should be explained that the author of this sentence is the wife of Dr. Guebhard. She is an advanced advocate of women's rights, yet condescends to thus express her opinion that a woman may do good work tending the sick as well as in speaking in Parliament.

PLAIN SPEAKING.—It is supposed to be one of the most prominent characteristics of the Americans, that they say what they mean in downright language. Certainly Dr. Gaillard Thomas did not mince matters in addressing the graduate nurses of Bellevue Hospital, when he gave them the following forcible advice:—"Do not, pray do not, get up relations of maudlin sentimentality with your patients and their families, however strong the temptation to do so may prove. Do not permit yourselves to believe, however often it may be told you by grateful employers, that your magical powers have saved from the grave some loved one, for whose life neither money nor thanks nor devotion can repay you! You are employed to do a certain work, do it to the best of your ability; you are well paid for it; be content; go on repeating the process and you will be happier in the end, more independent, and more dignified, than all the simpering sentimentality of an unnatural, short-lived and nonsensical devotion can make you."

THE SANITARY COMMISSION.—All who are acquainted with Bret Harte's quaint yet pathetic line entitled "How are you Sanitary?" must have desired to know more about the Sanitary Commission during the Civil War in America. Like many a great philanthropic movement it was started by the women, who at the beginning of the war formed themselves into a Relief Committee, and scraped lint and collected comforts for the wounded. The exigences of the campaign soon, however, got beyond their power to relieve, and the women sought the counsel of clergymen and doctors, who in their turn conferred with the Secretary of War. The result of the conference was the establishment of the Sanitary Commission, which undertook the complete charge of all sick soldiers and all wounded in battle. Hospitals were established wherever it was necessary, and thousands of nurses were enrolled, of all denominations. From the nurses trained by Lutheran charities alone two thousand were taken to work for the Sanitary Commission. And in this connexion the efforts of Miss Clara Barton in relation with the American Association of the Red Cross must always be applauded.

THE HAMILTON ASSOCIATION.—The *Daily News*, which always shows an interest in nursing matters, has devoted a paragraph to male nurses. Most people, it says, are accustomed to look upon nursing the sick as an essentially feminine occupation, and in the great majority of cases they are right. Every now and then, however, there are instances in which the strength and decision of a man would be even more beneficial than the gentle tenderness of a woman. Male nurses are almost a necessity for the proper care of a strong man under the frenzy of delirium, and they are highly desirable in some other forms of sickness. Nevertheless, a properly-trained nurse is a great rarity. Excellent women nurses can be easily got from any well-known institution, and no one would be much at a loss to know where to go in order to find them. With men nurses the case is different. There are few of them, and very few people would know where to procure the assistance of one in a sudden emergency. In giving particulars of where the male nurses of the Hamilton Association were sent last year, the *Daily News* implies that several were on duty at Guy's, whereas there was only one there for a short period. The association has the greatest demand amongst private cases, gentlemen who are used to valets not appreciating the thought of a woman's ministration when they are ill.

A SMALL-POX EXPERIENCE.—In the half-yearly number of "St. Margaret's Magazine," which has lately appeared, there is an account given of the experiences of two of the East Grinstead Sisters who went to Coleford during the small-pox epidemic last March. There is a graphic piece of writing which describes the burial of a patient, a very big man. The coffin was left in the garden of the temporary hospital, and the sisters carried it into the house, and then carried the corpse down stairs and put it in. A truck covered by a white sheet was used instead of the ordinary hearse, and on this the coffin was placed. Two men pulled the truck in front and one pushed behind, the two sisters were the only followers, but the sanitary inspector walked in front strewing disinfecting powder on the ground. No wonder such a gruesome funeral was watched with fearful glances. It speaks volumes for the sisters that they were able before they left to become friendly with the people, and banish the suspicion with which they were first regarded.

VOLUNTEER NURSES. — Some time ago Surgeon-Major Evatt called attention to the defective arrangements of the medical department of the Volunteer service. He wrote very strongly on the point, saying that in case of an invasion, the utter absence of any provision for their sick and wounded would so hamper the volunteers as to make them practically useless. Since then several steps in the right direction have been taken, the medical students being often enthusiastic over their ambulance drill, but the only volunteer nurses are those lately enrolled at Manchester. There must be plenty of women who have had six months or more hospital training, and are now in private life, who could sacrifice the time and labour necessary to make themselves useful should an invasion ever occur. It would not be very hard work to acquire some knowledge of the ambulance arrangements of an English army corps in case of war; to learn what are stationary hospitals, and what are base hospitals; and what are the duties of an army or navy sister. In fact, every nurse should be familiar with the outline of ambulance arrangements, so that should she wish to volunteer at any time, she could give good reason why she should be chosen before others. We all know how terrible were the sufferings endured in the Crimea through the lack of proper nursing arrangements, and though since then Red Cross Societies have been widely organized, much is far from perfect. France thought all had been done in the way of necessary ambulance knowledge before the Franco-German war; yet so late as 1870, after the battles of Worth and Suly, the wounded lay in hundreds on the ground for two days, without either food or care. The time of peace is the time to prepare for war, and it is a pity some person in authority does not take up the subject of volunteer nurses, and never drop it till it becomes a fact.

### Original Articles.

### A NURSE'S PROGRESS.—FROM APPLICATION TO CERTIFICATION.

### PAPER I. APPLICATION.

As every boy is supposed at some period of his life to wish to be a sailor, so every girl when she is in her teens decides she will be a hospital nurse. After much hesitation, many of these girls go the length of applying to some hospital, and by return of post comes a large blue envelope, full of rules and forms, and the dismayed applicant learns that there are a hundred reasons why she cannot immediately become a nurse. She has to get the consent of her parents, she has to be a certain age; indeed, there is scarcely a single necessary qualification which this young applicant fulfils. And apart from the demands made by the hospital authorities, there are many requirements of mind and character, without which it is hopeless to expect to become a good nurse. These requirements may be summed up as follows: A retentive memory, quick observation, habits of cleanliness and obedience, absolute unselfishness, a calm cheerful mind, and an enthusiastic love of nursing. A good memory and powers of observation are greatly the result of training, but without a certain natural aptitude they cannot be developed. Habits of cleanliness and obedience, if not acquired at home in early youth, will ever mar the character of a nurse. A spoilt child seldom grows into a good woman, and the influence of home discipline is the very best preparation for hospital life. Unselfish a nurse must ever be, for if she neglect her patient for one moment from consideration for self, she has deliberately failed in her duty, and is no longer worthy to be entrusted with a responsible post. As for a love of nursing, that is to a certain degree to be found in most women, but often disappears before the trials of ward-work. Those who take to this profession from a passing fancy, or because they are too undisciplined to live peaceably at home, or because it is more genteel than domestic service, are not actuated by what we call enthusiasm, and will never make good nurses.

Having a moderate amount of intellect and virtue, then, and a firm resolve to adopt a nurse's life, the next step is to apply to a hospital. There are always some doubts as to which hospital to choose: to enter a thoroughly good training school it is often necessary to wait a year or eighteen months, and here comes in the temptation to take service in some inferior institution, or some children's hospital. Yet a good nurse is only made by good training, and even from the monetary point of view it will pay better to wait awhile and work harder to obtain a good certificate, than to be content to take a second-rate standing. Some of the county infirmaries offer very good training without keeping their applicants long waiting, but the chosen hospital should contain at least a hundred beds, and be situated in a large town. With regard to the London hospitals, a High Churchwoman would probably apply to University, King's College, or Charing Cross; a Roman Catholic or Dissenter would apply to the London, which is the most unsectarian of the hospitals. At Guy's the lady pupils do no rough work, but they pay a guinea a week and provide their own uniform. After a year, or two years, they are generally made sisters. At St. Bartholomew's the probationers are all on the same footing, and do a great deal of rough work. They are bound for three years before they get their certificate. At St. Thomas's the probationers are made extremely comfortable, and at Middlesex the ordinary nurse is more likely to be made a sister than the lady-pupil. The points to consider before applying to a hospital are: (1.) Whether you wish to train one, two, or three years for your certificate. (2.) Whether you wish to pay for being taught or to be paid for learning. (3.) Whether you wish hard work and wide experience, or a quiet time and less knowledge.

Having decided these points and chosen your hospital, the next step is to apply to the matron, who will send you a copy of the regulations, a time-table giving the length of working hours, and a form containing some dozen questions to which you have to write answers. This form must be filled up with the greatest care and accuracy, and there must be no holding back of facts, or equivocal answers. What must be the matron's opinion of a woman who, in answer to the question "Are you strong and healthy and have you always been so?" simply answers "Yes," though she has suffered from rheumatic and scarlet fever, both of which constantly leave permanent mischief behind them? In giving the addresses of two persons as references, it is as well to choose professional people. A doctor or a clergyman who has been acquainted with you all your life is the very best reference possible. The following is a list of the questions generally asked by a training school, and which should be immediately answered in the candidate's own handwriting, and returned to the matron:—

1. Name in full and present address of candidate.
2. Are you a single woman or widow?
3. What has been your occupation?
4. Age last birthday, and date and place of birth?
5. Height?
6. Where educated?
7. Are you strong and healthy, and have you always been so.
8. Are your sight and hearing perfect?
9. Have you any physical defects?
10. Have you any tendency to pulmonary complaint?
11. If a widow, have you children? How many? Their ages? How are they provided for?
12. Where (if any) was your last situation? How long were you in it?
13. The names in full and addresses of two persons to be referred to. State how long each has known you. If previously employed, one of these must be the last employer.
14. Have you read and do you clearly understand the regulations?

The last question is omitted by many schools, and yet is decidedly necessary, for many a nurse has entered a hospital without ever having studied the time-table sent her fully enough to comprehend that she is often on duty for twelve hours at a stretch.

Having now applied to the chosen hospital, it remains to the candidate to use well the period of waiting by preparing for the fray. Probably the uniform has to be made, and a good stock of under-linen must be obtained. At least double the amount required at home will be needed in the hospital, and collars and cuffs must be numerous. Everything must be plainly marked and strongly made, for a nurse has very little time to devote to her own wardrobe, or to worrying over garments lost at the wash. Woollen stockings of a soft, light texture should be procured, and two pairs of easy, noiseless house-shoes. These last are none the worse for being a size too large. If the aprons are made with large pockets, there will no necessity to wear a jingling chatelaine. During the personal interview with the matron, it is as well for the candidate to ask what text-book on nursing she had better get, and, having procured it, she should at once read it carefully. If there are any ambulance classes being held in the neighbourhood, they should also be attended. A woman is spared many an awkward position by having a slight acquaintance with nursing appliances before she enters the wards. It is so difficult for a sister to remember the absolute ignorance of a new probationer, that she constantly sends her to fetch a spatula or a hypodermic syringe, when the poor probationer has not the least idea what either of these articles are like. If going to a hospital where each nurse has a room to herself, the candidate can pack a few photographs and treasures to brighten her room; but if going where the nurses are crowded together in the attics, the less luggage taken the better. The portmanteau, however, should always contain the following extras: Some scented soap, a tea-cup and saucer and spoon, a bottle of eau-de-cologne, a box of biscuits, and a packet of cocoa. These few things will seldom come amiss.

### British Nurses' Association.

We are indebted to Miss Corvan, Lady Superintendent of St. George's Home for Nurses, Sheffield, for the following account of the meeting of the British Nurses' Association, held there. It seems, however, that there was some irregularity in the last proceeding, the nomination of Miss Cadbury as secretary for Sheffield and the district. The laws of the Association provide that the appointment of all local secretaries shall rest solely with the central authority in London: "A meeting in connexion with the British Nurses' Association was held at St. Georges' Home for Nurses, Sheffield, on Saturday evening, July 28th. Nurses from the Infirmary Public Hospital, West Street, Jessop Hospital for Women, Children's Hospital, and St. Georges' Home, were present; also Miss Cadbury (Matron of the Public Hospital), Miss Corvan (Lady Superintendent of St. Georges' Home). Miss Booth (Matron of the Jessop Hospital), Miss Spencer (of the Infirmary), and Miss Pounthey (of the Children's Hospital), were out of town; and Miss Armstrong (Lady Superintendent Nurses' Home, Glossop Road) wrote to say that, owing to a previous engagement, she could not be present, but she took a great interest in the Association. Miss Cadbury, who presided, gave a most interesting account of the meeting in Birmingham, and read a great part of Miss Wood's address. The following resolutions were proposed and carried unanimously: 1st. That the time has come for nurses' registration, and that a Royal charter is desirable for the purpose of carrying this out. 2nd. That the examinations of nurses should be carried on from one centre. 3rd. That there should be three examinations, one at the end of each year —that at the end of the first year to be on general subjects, and not too difficult. An animated discussion followed, in which the nurses from the various institutions took part. Many questions which the future scheme must answer were asked. For example, how many chances of passing would be allowed? Would there be certain standard books to study? Could a nurse take up special subjects? There was also a question raised as to what examinations private nurses should pass, as the difficulty of studying for them would be so great after the first year, when they have left the hospital. At the conclusion of the discussion it was proposed by Miss Corvan, seconded by Miss Booth, and passed unanimously: That Miss Cadbury be appointed secretary for Sheffield and the neighbourhood."

### Everybody's Opinion.

[*Correspondence on all subjects is invited. No communications can be entertained if the name and address of the correspondent is not given, or unless one side of the paper only be written on.*]

### DISTRICT NURSING.

Will you kindly allow me to make a few remarks on the article on District Nursing published in the Nursing Supplement of the 14th inst.? I do not know anything about the rules of the Metropolitan and National Nursing Association; but I have worked as district nurse in six large provincial towns and one country parish, so that you will believe I know something of the work.

First, as to the hours of work. All the Homes that I have worked with commence work at nine in the morning, returning about one; rest in the afternoon; out again at six, and come in when their work is done—sometimes as late as ten, but more often at nine. The work is pretty much the same as in the wards of a hospital. We keep charts of the temperature, pulse, and respiration; wash the patients, attend to their backs, make and change their beds, apply poultices or dressings, brush out throats, &c. Very often a doctor will have an operation to perform, or a patient to examine, or may wish to see a wound dressed; in any of these cases the nurse should be asked to meet him.

As to the statement made that a district nurse has always a majority of chronic cases on her books, this is not always true. My present district is a very large one, which I have worked in for four years, and my class of cases has been equal to any that can be found in the wards of a hospital. For instance, amputation of arm at the shoulder, lithotomy, herniotomy, colotomy, empyæmia, and numerous smaller operations. The medical cases are equally good. A district nurse must possess both skill and confidence in herself, as she is often called upon to acc very promptly, or life would be extinct before a doctor could arrive.

About the "unlearned committee" I cannot say anything, as I have never had the misfortune to work under such. As you say, a district nurse has her conscience to guide her; and, with a good training, what can she need more? R. C.

Manchester.

### OLD NURSES AND NEW.

Now that nursing has become the fashionable way in which a lady may earn a livelihood, the question naturally arises as to what is expected of the nurse of to-day. A great deal has been said on the subject; too much perhaps for the good of the profession. Nearly all the papers one takes up contain something about the nursing world. It may be a breach of promise case, or a sensational elopement, or an act of heroism; but a nurse is always the central figure. The nurses of twenty years ago were, for the most part, women that would now fill the place of ward-maids, uneducated perhaps, but kindly well-meaning people, who had seen the dark side of life. But since Miss Nightingale revolutionized the nursing world "the nurse" is the choicest and fairest of the world's characters—a cheerful, well-educated Christian, beautiful both in mind and body. But, after all, it is only an ideal, and because they do not attain this standard, but prove themselves only ministering women, and not the ministering angels they were expected to be that they have a shower of complaints falling upon their heads. It should be remembered that nurses have to be trained. They do not come into an hospital for the first time already trained, however capable they may think themselves, and they will have their share of ups and downs before they become master of their profession. I greatly appreciate the correspondence in The Hospital, and think it would be well if the nurses took more interest in it.

Cicely.

### SEASIDE CONVALESCENT HOMES.

Almost every week I see answers to questions on all sorts of subjects. Could you tell me the address of a convalescent home at the seaside, where a non-subscriber could send a man for a few weeks, paying a moderate weekly sum. The man in question is a hard-working man—wooden ware—and has got thoroughly run down. He has been some weeks in this hospital, and is much better. The squire here has tickets for Blackrock, Brighton, but they are all promised. He would send him away if we knew of some seaside home where they could take him in soon, as he is anxious to get back to work, though hardly strong enough.

Emily F. Campbell, Nurse-Matron.

Cottage Hospital, Chesham, Bucks.

[Miss Campbell should apply to Mrs. Marshman, 4, Ladbroke Square, Notting Hill, London, who might be able to arrange for the man's immediate admission for an adequate payment to her Convalescent Home, Brighton.—Ed. *T. H.*]

### FREE TRAINING IN MIDWIFERY.

Will you kindly inform me where I can receive free training in midwifery? I am 29 years of age, and have been three years in a small children's hospital, and six years a private nurse. I have been at St. Pancras Infirmary as night nurse for five months, but find I cannot go on with night work, so have been advised to write to you. I have been to the Workhouse Nursing Association, but they have no vacancy till next April, and as I have to help support my aged parents, I cannot afford to wait so long. If you could kindly inform me of any hospital where I could get free training in midwifery I should be very glad. I am disappointed to find, after so many years of nursing, that I am not recognised as a "trained nurse." This is why I want to become a certificated midwife. A. M.

### Honours and Presentations.

[Information for this Column should be addressed to THE EDITOR, The Lodge, Porchester Square, W.]

THE *Worcestershire Chronicle* of the 4th inst. states: The house surgeon of the Worcester Infirmary, Mr. T. P. Gostling, having received notice from the committee to leave the infirmary shortly, the nurses of the institution determined to make a presentation to him as a mark of the universal respect and esteem in which he is held by all connected with the infirmary, and as an expression of their regret at his departure. The presentation, made by Nurse Naergër, consisted of a pair of handsome bronze ornaments, and an address, which was signed by Prudence T. Naergër (head nurse), Emma Shepherd, Nellie Atkinson, Frances Place, and Nellie Hyde. The names of the probationary nurses followed. Mr. Gostling, in happy and appropriate terms, expressed his pleasure at receiving the presentation, which he should look upon as a souvenir of the pleasant time he had spent in that institution.

### QUITE SO!

OUR contemporary, the *Philanthropist*, for August, has the following pertinent paragraph: "Mr. Burdett has scored one against the British Nursing Association. When it was proposed that the public should take such an interest in the National Pension Fund for Nurses as to help it with subscriptions, there was a great and indignant cry against the suggestion so humiliating to independent people like nurses. Their leaders were very lofty in their wrath. They characterised the suggestion as "insulting." But now the Princess Christian suggests that the British Nursing Association is in need of public subscriptions to carry out their laudable scheme. And Mr. Burdett wants to know why. Is registration such a costly process? Or is the present registration scheme too ambitious? that the public should subscribe to help a pension fund for nurses one understands; but what funds does a scheme of registration require?"

### Appointments.

[It is requested that successful candidates will send a copy of their applications and testimonials, with date of election, to THE EDITOR, The Lodge, Porchester Square, W.]

EVESHAM SANATORIUM.—Miss Adkin has been appointed superintendent of this sanatorium. Miss Adkin was partially trained at the London Hospital for a period of six months, and worked for some time for the Stratford-on-Avon Nursing Institution. Lately she has had charge of the Copham Hill Sanatorium for Infectious Diseases at Stratford-on-Avon, which post she is leaving to take up the work at Evesham where Miss Mawer left it. We hope Miss Adkin will find a warm welcome.

ERRATUM.—The number of nurses at the Middlesex Institute was stated in our issue of the 28th ult. to be 24, but we have since learnt that there are but 11 there at present.

### Notes and Queries.

TO CORRESPONDENTS.—1. Questions may be written on post-cards. 2. Advertisements in disguise are inadmissible. 3. In answering a query please quote the number. 4. If a private answer is desired, a stamped addressed envelope must be forwarded.

(40) *Nurse Training in Military Hospitals.*—Would you kindly send me the address of the institution at Netley where women are trained for nursing in military hospitals?—FLORENCE M. BEST, Oxford.—Apply to the Matron, Royal Victoria Hospital, Netley, Southampton.

(41) *F. F.* would be obliged if Mr. Editor would tell her through THE HOSPITAL where the Red Cross quilts are to be had.

(42) *The Nursery Yacht.*—Miss Baster writes from the Royal Berks Hospital, Reading—I shall feel greatly obliged if you can tell me where I can obtain particulars of the "Nursery Yacht," or rocking boat mentioned in THE HOSPITAL, of July 21st, as patented by Dr. Illingworth.

**Answers.**

(37) *Massage.*—Apply to Mr. M. E. Cope, Masseur, 2, Roebuck Terrace, Thorpe Road. Forest Gate.

(39) *Private Nursing in New York.*—There is an "Association of Trained Nurses" in New York, the address of which is, I believe, West Thirty-fourth Street. I understand that the nurses pay 15 dollars down, and take their own fees, these varying from 10 to 20 dollars per week—£2 1*s.* 8*d.* to £4 3*s.* 4*d.* These particulars were given me by the lady-in-charge at Thirty-fourth Street. I do not remember any private nursing institution; and whether such are sent out from the hospitals I am not sure.—L. BOWYER, 9, Lupus Street, S.W.

### Vacancies.

The following vacancies are announced:—

**Nurses.**

Beaumaris District; Midwifery, qualification and knowledge of Welsh indispensable.

Devon and Exeter Hospital, Exeter; Two, £27.

Harrogate Bath Hospital; £30.

Hospital for Consumption, Brompton; £25.

Staffordshire General Infirmary, Stafford; Two.

West Sussex Infirmary, Chichester; Several.

Kingston Union, Surrey; £40.

Kent Nursing Institution; Several.

Victoria Home for Nurses, Bournemouth; Several.

Royal National Hospital for Consumption, Ventnor, I.W.

Kingston Nurses' Home, Hull; Several.

St. George's Home for Nurses Sheffield; Several.

Swansea and South Wales Nurses Institution; Several.

Cancer Hospital (Free), Brompton, S.W.; Several.

**Matron.**

Bradford Infirmary, Superintendent of Nurses; £60.

**Wardmaid.**

Cottage Hospital, Wallasey, Cheshire.

**Probationers.**

West Sussex Infirmary, Chichester; Several.

Cottage Hospital, Wallasey, Cheshire.

Royal Hospital for Diseases of the Chest.

